# Investigation of the Mechanical and Microstructural Properties of Masonry Mortar Made with Seashell Particles

**DOI:** 10.3390/ma16062471

**Published:** 2023-03-20

**Authors:** David O. Nduka, Emmanuel T. Akanbi, Daniel O. Ojo, Timilehin E. Babayemi, Kayode J. Jolayemi

**Affiliations:** 1 Department of Building Technology, College of Science and Technology, Covenant University, Km 10 Idiroko Road, Ota 112233, Nigeria; 2 Department of Civil Engineering, College of Engineering, Covenant University, Km 10 Idiroko Road, Ota 112233, Nigeria

**Keywords:** cement mortar, seashell particles, seashell-blended mortar, microstructure, mechanical properties, sustainability

## Abstract

In order to study the mechanical and microstructural properties of masonry mortar, combined particles of cockle and scallop seashell wastes were incorporated and analysed through destructive and non-destructive tests. River sand was replaced with the combined seashell particles (SPs) at seven mixes, viz., 0, 5, 10, 15, 20, 25, and 30% with a 0.5 constant water-to-cement ratio (W/C). A mortar mix design of M4-type of BS EN 1996-1-1 was adopted with a target compressive strength of 5.17 MPa at 28 days. The physical, chemical and mineralogy properties of the SPs were analysed through BS standard sieving, X-ray fluorescence (XRF), scanning electron microscopy (SEM), and X-ray diffraction (XRD) methods. The hardened SP-based mortars were subjected to direct compressive strength, rebound hammer, ultrasonic pulse velocity tests, and nonevaporable degree of hydration analysis. The XRF, SEM, and XRD analysis results of the SPs showed over 86% calcium oxide content, irregular and needle-like particles, and hydroxyapatite/calcium silicates, respectively. The direct compressive strength and the non-destructive test results revealed that up to 30% sand replacement with SP in masonry mortar, an improvement of 45% compressive strength could be attained over the control sample. The nonevaporable water method of the degree of hydration analysis showed that after 28 days, hydration increased considerably for the SP-blended mortars over the control, especially the SPM-30 with 30% sand replacement. Therefore, the study concludes that the investigated SPs in blended masonry mortar could benefit an eco-friendly environment and conservation of natural resources.

## 1. Introduction

The heightened interest in using cement composite materials in the architecture, engineering, and construction (AEC) industry has spurred the demand for developing eco-friendly mortars. One of the cardinal reasons for this is linked to the damaging environmental impact derivable from using mortar or concrete-making materials, including sand and Portland cement, PC [1,2]. Increased sand consumption results in the exhaustion of natural reserves, and unrestrained sand mining could result in landscape and ecosystem destruction, as well as water, soil, and air contamination [3]. Measures to control mining operations in many regions have seen authorities imposing restrictions on the mining of sand via taxation and banning mining on some sites [4]. Researchers have consciously attempted to incorporate waste products as a partial replacement for cement and aggregates in cement-based composite products. Incorporating waste products into the AEC industry is mainly for environmental and natural resources preservation [4]. Robust benefits proposed by Bamigboye et al. [5] include a decrease in material logistics and removal costs and an increase in landfills’ design life and capacity. In some cases, lower material costs compared to unexplored materials. Thus, the practice may be more beneficial if transportation, storage, and processing costs are lower than those of conventional materials.

Prior Portland cement studies investigating cement-based composite products have indicated environmental degradation, by-product disposal, the harmful release of carbon dioxide (CO_2_) and depletion of natural resources as the major concerns of cement manufacturing [6]. For instance, Peys et al. [7] attributed about 3 Gt/year of CO_2_ emission and approximately 8% of total anthropogenic CO_2_ global emission to the cement industry. Moreover, limestone, a non-renewable natural resource that accounts for about 85% of cement raw meal, is depleting at a high rate causing ecological problems [8]. In addition, issues surrounding Portland cement on cementitious products, including shrinkage during curing, cracking, structural or durability deteriorations, limited resistance to certain chemical ions, and destabilisation under elevated temperature and pressure have been identified [9]. Consequently, all such awful characteristics will have a deleterious impact on the wholeness of cement-based composite products and may create pathways for ion migration in the hardened cement matrix. Nevertheless, to achieve a superior quality of mortar or concrete, treated aquaculture seashells serving as a replacement for cement, sand, and granite have been found in recent empirical studies [5,10,11,12,13]. Seashell is a hard, protective outer layer produced by marine organisms. Other animals have usually eaten the soft part, thus the empty shell.

Seashell is composed mainly of aragonite and calcite with higher densities and strength than limestone powder [12]. Bamibgoye et al. [5] described seashell as a notable filler in cement-based products with over 90% calcium carbonate comparable with limestone, serving as the main raw meal for limestone Portland cement. It is needle-like in powdered form and has a specific gravity range of 1.85 to 2.73 and nearly zero moisture content [4]. Bamibgoye et al. [5] adduced that a seashell’s chemical compositions are affected by the collection region. When used as a cement substitute in cement-based products, it forms a structured mesh and smaller pores, which may lead to increased compressive and flexural strengths [3] and reduced permeability and porosities with over 15% by weight content in a cementitious matrix [5]. It also provides insulating properties to cement-based products [12]. Classification of seashells utilised in cement-based products, according to Eziefula et al. [4], are mainly bivalve shells and gastropods of mollusc shellfishes. The authors estimated molluscs production to be around 16 million tonnes amounting to 22% of total aquaculture production yearly. In bivalve mollusc species, clam (ark-shell and cockles) is 33%, 31.3% represent oysters, 12.1% are mussels, and 10.9% are pectins and scallops. Around 2.8% are abalones, winkles, and conchs. Therefore, the relative availability, chemical oxides content, and mineral phases of seashells signpost their relevance in cement-based products.

Similarly, Suarez-Riera et al. [14] estimated the annual European Union and Chinese seashell waste production to be around 600,000 and 10 million tonnes, respectively. Industries, including music, pharmaceutical and medical, art, and ornament, have found the waste useful [15]. Irrespective of the usage, large quantities are dumped or disposed of in landfills with attendant environmental menaces of offensive odour and a distracting visual environment [12,15]. Long-term storage can result in the biological degradation of salts into gases such as hydrogen sulphide, ammonia, and amines [4,14,15]. Nevertheless, incorporating the waste in simple concrete structures such as residential homes, septic tanks, soakaway pits, grave slabs, pavement slabs, and drainages, however, demonstrates the use of seashells as a substitute for aggregate by local builders in Nigeria [4,5,10]. 

In driving the utilisation of seashell wastes in cementitious products, reviewed papers [3,4,10,15,16,17] and experimental works [5,11,12,13,14,15,18,19,20,21,22,23] have studied the feasibility of incorporating various types into cement-based composite products. Mo et al.’s [3,16] and Eziefula et al.’s [4] review studies concluded that using a seashell as a substitute for aggregate in concrete reduces the physicomechanical properties of seashell-based concrete while up to 50% replacement level can develop a concrete applicable for non-structural use. Similarly, Bamigboye et al. [5] and Tayeh et al. [15] found that including calcined seashell ash as a cement replacement in a cementitious matrix produces concrete with improved strength at a later age and minimises the ions migration in concrete at a higher cement replacement level. 

In experimental works that added waste seashells in cementitious materials, Lertwattanaruk et al. [18] empirically studied four types of seashells (short-necked clam, green mussel, oyster, and cockle) in ground form for cement replacement in masonry and plastering mortars. The author’s findings showed that seashell-based mortars attained satisfactory strength, less shrinkage, and lower thermal conductivity later than the plain mortar with Portland cement. Safi et al. [19] investigated the usefulness of seashells in a self-compacting mortar. The authors replaced the sand with seashells up to 100%. Their results indicate that 100% sand substitution with seashells best improved the flowability of the tested self-compacting mortar with a slight reduction in compressive strength and elastic modulus values. Moreover, the microstructure of their tested hardened mortar samples was observed to have good adhesion between calcined seashell powder and cement paste. Similarly, Wang and Liu [20] experimentally found that seashell powder in cement paste is capable of refining the microstructure of the nanostructure of hydrated C-S-H gel than limestone powder. This reaction behaviour is attributed to the aragonite mineral phase content of seashells. Aragonite is an amorphous crystal type of calcium carbonate found in maximum content in seashells. The use of Babylonia *areolata* seashell of Southern China was experimented with in foam concrete applied in an embankment project [21]. The form seashell partially and wholly replaced coarse aggregate in concrete mixtures. At the same time, engineering properties of flowability, compressive and splitting tensile strengths, drying shrinkage, water absorption, and rapid chloride ion permeability tests were performed. The study’s findings showed that incorporating seashells in foam concrete improved the workability and durability performances of the tested samples. 

Tayeh et al. [23] utilised the *glycymeris nummaria* bivalve calm specie of seashell calcined up to 1000 °C to produce a seashell powder. The powder was blended in concrete to produce a target compressive strength value of 25 MPa at a fixed W/C of 0.65. A replacement level of five steps, viz., 5, 10, 15, and 20% were adopted. The study concluded that 5% cement replacement with seashell was best for mechanical and durability dimensions of sustainable seashell-blended concrete. Similarly, Bamigboye et al. [5] crushed the *senilla senillis* (bloody cockle) type of seashell of southwestern Nigeria as a coarse aggregate replacement for 25 MPa compressive strength concrete using a constant 0.5 W/C ratio. The authors produced seashell-blended concrete replaced at 10, 20, 30, and 40% while investigating for fresh and mechanical property tests. Their findings revealed that 10 and 20% coarse aggregate replacement with seashell could attain M20 grade concrete for structural use. Hasnaoui et al. [11] tested queen scallop seashell type from northwest France in a blended alkali-activated seashell waste mortar. After the seashell’s mechanical and chemical product characterisation, a mortar was produced and activated at 2.5, 3, and 3.5% of a seashell. The author’s findings showed a promising result of improved mechanical and durability properties of the seashell-blended mortar. An examination of the microstructure of hardened seashell-blended mortar via scanning electron microscopy image showed good compatibility of seashell and aggregates. 

In fireproofing building material application, seashell waste (northern scallop and Mediterranean mussel, Spain) was studied by Peceño et al. [22]. The researchers replaced gypsum at four steps of 0, 40, 60, and 80% with seashell and characterised raw seashell’s physical, mechanical, and insulating properties in the blended gypsum mixture. Their finding indicated that a technically sound fireproofing panel was manufactured with up to 60% gypsum replacement with a seashell. Sangeetha et al. [13] empirically demonstrated the impact of calcined seashell ash and particles as a partial cement and aggregate replacement in M25 grade concrete. The authors investigated compressive, flexural, and splitting tensile strength mechanical properties after 7, 28, 56, and 90 days of hydration using a constant 0.5 W/C ratio. Their study found that 5% cement replacement with seashell powder and 10% aggregate replacements with seashell coarse aggregate provided the optimum for an eco-friendly seashell concrete. Moreover, Ahsan et al. [12] utilised seashells obtained from the Arabian sea coast, Karachi, Pakistan, to evaluate the mechanical properties and degradation effect of elevated temperatures on a seashell-modified high-strength concrete. The sand was replaced with seashells at a maximum particle size of 0.15 mm. The investigator’s findings revealed that seashell-modified high-strength concrete performed better than the control sample in mechanical properties and at high-temperature degradation exposures. 

Consequently, due to the availability of mollusc species of seashell in Nigeria, accounting for about 30% of annual fishery capture and aquaculture data between 1980–2017 [5], there is the feasibility of attempting the incorporation of seashell waste as a substitute in a sustainable cement-based composite product in Nigeria. Therefore, this study investigates the mechanical and microstructural behaviours of scallop and cockle seashell particles (SPs) in the blended masonry mortar of a designed 28 days compressive strength of 5.17 MPa. Scallop (53.70 CaCO_3_ content) and cockle (51.56–54.24 CaCO_3_ content) seashell species [4] were combined as a replacement for sand due to their close calcium carbonate content. The cement and cement–lime mortars specified by BS EN 1996-1-1 [24] classify masonry mortar as M4 type signifying a volume proportion of 1:0–1:5–6. The compressive strength is denoted by the letter M, followed by the compressive strength in MPa. The study is significant for understanding scallop and cockle seashell types’ potential as a raw material in cement-based products. This study will assist in creating a cleaner environment, lessen pollution caused by the aquaculture industry, and reduce the ill effect on the environment arising from Nigeria’s continuous and unending quarrying and mining process. 

## 2. Materials and Methods

### 2.1. Outline of Experiment

As shown in Table 1, an experimental plan was developed to investigate the material properties of seashells particles (SPs) and their effect on the mechanical and microstructure properties of the Portland cement (PC) mortar matrix. To determine the composition of SPs, X-ray fluorescence (XRF, Bruker AXS S4, Explorer, Karlsruhe, Germany), scanning electron microscopy (SEM, Phenom ProX, PhenomWorld Eindhoven, The Netherland), and X-ray diffraction (XRD, Rigaku Miniflex 600, Washington, DC, USA) were utilised.

After knowing the material properties, SPs were incorporated at 0 to 30% by weight of aggregate (b_woa_) contents at a 5% steps interval. The compressive strength using destructive and non-destructive tests was employed to determine the strength and integrity of the hardened SP-based mortar samples. The degree of hydration dynamics of the same mortars was analysed using the nonevaporable water technique. In addition, the quantitative and qualitative analyses of selected hardened SP-based mortar samples’ mineralogical phases were characterised using the XRD method.

### 2.2. Materials

**Figure 1:**
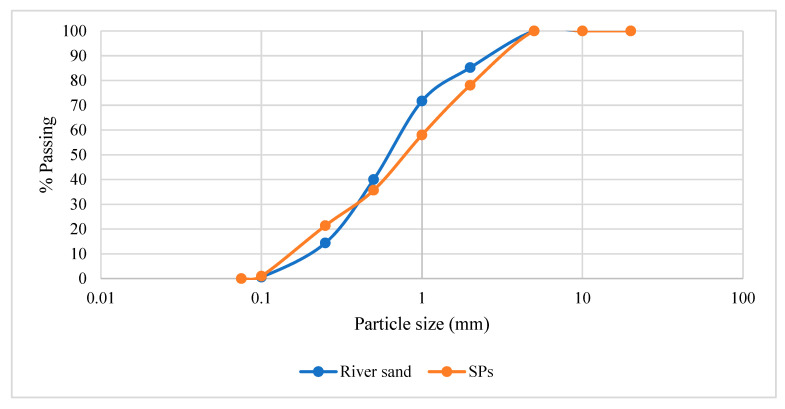
Particle size distribution of aggregates.

The materials used in this research were Portland cement as per BS EN 197-1 [25] and NIS 444-1 [26], river sand as per BS EN 13139 [27], and water as per BS EN 1008 [28]. The raw seashells (scallop and cockle species) were obtained from Badagry seashore, Lagos state, southwestern Nigeria. They were handpicked from the beach, washed thoroughly under running water to avoid impurities, and oven-dried for 24 h at 150 °C. After, the seashells were reduced to fine aggregate sizes using the Los Angeles abrasion machine and later calcined to 600 °C for calcium oxide (CaO) activation. The thermally decomposed granules were sieved through a 2 mm BS standard sieve for a sand replacement for mortar application. The preparation of seashells as a substitute for fine aggregate followed the recommendations by Eziefula et al.’s [4], Bamigboye et al.’s [10], and Tayeh et al.’s [15] reviewed papers. The sand and SP’s particle size distribution (PSD) sizes were measured via the BS EN 933-1 [29] standard sieving method presented in Figure 1. The chemical compositions of the SP analysed through the XRF technique are shown in Table 2. Moreover, the physical properties of the river sand and SP sizes used in the present study are highlighted in Table 3.

### 2.3. Specimen Preparation

Cubic mortar samples of 40 mm cubes were prepared for destructive and non-destructive compressive strength testing under BS EN 1015-11 [30], BS 1881-202 [31], and BS EN 12504-4 [32], respectively. Using nonevaporable water, a study of the degree of hydration of the tested samples was conducted. According to BS EN 1996-1-1 [24] volumetric mortar classification, Table 4 displays the mix design of M4-Type mortar specimens adopted in the study of a target compressive strength of 5.17 MPa at 28 curing days. The water-to-binder ratio (W/B) was maintained at 0.5, and SPs were added in 5% increments ranging from 0 to 30% b_woa_. The mortar classifications were labelled SPM and contained SPs. For example, the mortar containing 5% SPs was designated as SPM-5. After casting, each sample was cured at room temperature for 24 h. Until the experiment date, specimens were cured in water at 21 ± 2 °C after being removed from the mould. To characterise the hydration products, XRD techniques were applied to the 7- and 28-day-old remains of crushed control and SPM replacement mortar samples containing 30% PC. After truncating hydration with acetone for 24 h, 75 μm masonry mortar samples were XRD-analysed.

### 2.4. Casting, Curing, and Crushing

The cubes were cast in a mould with dimensions of 40 mm^3^. Twelve (12) cubes were produced for each percentage substitution of SPs as fine aggregate in the final product. In other words, eighty-four (84) cubes of 0%, 5%, 10%, 15%, 20%, 25%, and 30% SPs were substituted, respectively. This process resulted in the preparation of two hundred and fifty-two (252) cubes that were cast and vibrated. After 24 h, these same cubes were removed from the moulds and deposited in a curing tank at 21 ± 2 °C. Concurrent with curing cubes, crushing and other tests were carried out, and the resulting strengths were recorded at ages 7, 14, 21, and 28 days, respectively.

### 2.5. Test Methods

#### 2.5.1. Characterisation of Sand and Seashell

The BS sieving technique evaluated river sand and SP size distribution (PSD). In addition, XRF (Bruker AXS S4, Explorer, Karlsruhe, Germany), XRD (Rigaku Miniflex 600, Washington, DC, USA), and SEM (Phenom ProX, PhenomWorld, Eindhoven, The Netherlands) were used to examine the chemical oxides, mineralogical compositions, and morphology of the activated SPs. 

#### 2.5.2. Mechanical Properties

##### Direct Compressive Strength

Eighty-four (84) cube samples were prepared based on four curing ages and triplicate samples per age crush following BS EN 1015-11 [30] guidelines. The average triplicate sample value for direct compressive strength determination was done using a Model YES-2000 machine. The samples were tested after 7, 14, 21, and 28 days of curing following BS EN 1015:11 [30] specification. The compressive strength (*f_c_*) is calculated using Equation (1):

(1) $$f_{c}=\frac{F}{Ac}$$

where *f*_c_ is the compressive strength of the mortar mixture, MPa; *F* is the maximum load at failure, kN; *Ac* is the specimen area, mm. 

##### Schmidt Rebound Hammer Test

A digital Schmidt rebound hammer of Type-YD225E was used for this study and performed on all eighty-four (84) cube samples in line with BS1881-202 [31] protocols. The test was conducted on the mortar’s specimen at 7, 14, 21, and 28 days of curing. The rebound hammer test application on the prepared mortars is premised on identifying an acceptable margin of error, a link between surface hardness and compressive strength [32]. The rebound hammer index (RHI) provides an arbitrary indicator that rests on the device spring’s mass and energy storage capacity [33].

##### Ultrasonic Pulse Velocity Test

To evaluate the quality of the prepared mortar samples, this non-destructive experiment was modified from the BS EN 12504-4 [34] standard test method for pulse velocity through concrete. In this section, the Matest C369N Pocket Ultrasonic Pulse Velocity Tester was utilised on eighty-four (84) cube mortar samples measuring 40 × 40 × 40 mm^3^. After 7, 14, 21, and 28 days, ultrasonic pulse velocity measurements were taken. The transducer’s frequency is equal to 54 kHz. The transducer and receiver were installed on opposite sides of the cube. The test is conducted to determine the ultrasonic pulse passage time through the masonry material section [35].

##### Degree of Hydration Test

Utilising the nonevaporable water technique described by Nduka et al. [36], the degree of hydration of SP-based mortar samples was determined. For the purpose of determining the hydration growth tendencies of the mortars, fragments from 40 mm cube samples crushed after various curing days of 7, 14, 21, and 28 days were selected. Using the aggregate crushing value (ACV Test) mould and a 25 mm diameter bar as a mortar and pestle, approximately 5 g of each mix identification fragment at 7, 14, 21, and 28 days of curing was then appropriately milled. The sample was sieved, and a known weight of approximately 2 g was determined from the particles passing the 300 μm BS standard sieve. The sample was then oven-dried for 24 h at 105 °C ± 5 °C and weighed again to determine the amount of evaporable water, i.e., capillary water + gel water. The substance was then heated for 1 h at 950 °C and weighed (this measurement is used to determine the amount of chemically bound water). After that, all calculations were performed based on the ignited weight, yielding the following results:

The PC, SP and hydrated mortar paste’s loss on ignition (LOI) was determined using:

(2) $$\mathrm{LOI}\;(\%)=\frac{\mathrm{ARW}\;-\;\mathrm{IW}\;}{\mathrm{ARW}}\times 100$$

where ARW denotes as received weight and IW is ignited weight.

The hydrated mortar pastes’ W*n* (nonevaporable water) content was measured to assess the degree of hydration, as recommended in the literature [37]. The change in the mass quantity of the crushed paste at 950 °C and 105 °C is used to determine the degree of hydration (α) on the premise that 1 g of anhydrous cement generates 0.23 g of W*n*, thus W*n* is calculated using the formula below.

(3) $$Wn\;(\%)=\frac{\mathrm{DWP}\;-\;\mathrm{IWP}}{\mathrm{IWP}\;-\;\mathrm{LOI}}\times 100$$

where DWP is the dried weight of paste and IWP is the ignited weight of paste.

Finally, the degree of hydration (α) is then:

(4) $$\alpha =\frac{Wn}{0.23}\times 100$$

#### 2.5.3. Quantitative and Qualitative Analysis of Hydration Products

The crystalline phases and hydration products of finely ground and homogenised SP mortar samples were examined using XRD (K reflections). The 2–80° scan range with two-theta degree steps at 8.67 s per step was selected. Using the front-loading method, the powdered sample was placed in the middle of the sample holder and evenly distributed. The sample was then pressed flush with the sample holder using the glass slide. The sample holder was loaded laterally into the XRD, and an upward adjustment was made to ensure that the sample was in firm contact with the apparatus. 

## 3. Results and Discussion

### 3.1. Characterisation of Seashell

#### 3.1.1. SEM/EDX

**Figure 2:**
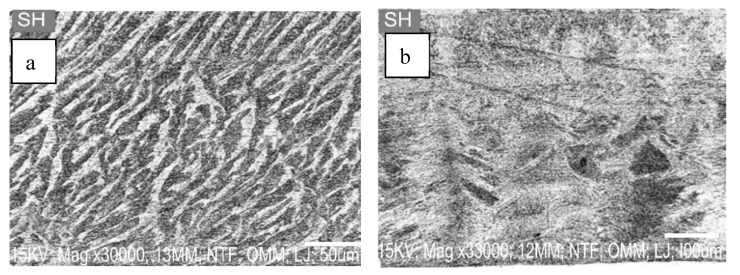
(**a**,**b**): The SEM images of SP.

Figure 2a,b show the SEM images of SPs used in this study when viewed at 3000 µm magnification of 50 and 100 µm settings, respectively. The SEM micrographs showed an irregular and needle-like particle size, with multi-layer shapes and flakiness conforming to Bamigboye et al.’s [10] review findings.

#### 3.1.2. XRD

**Figure 3:**
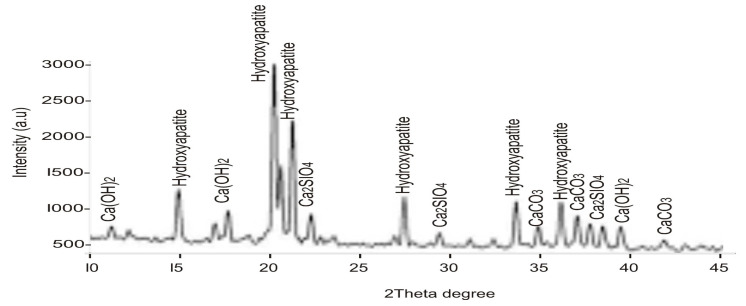
XRD pattern of SPs.

Figure 3 shows the diffractograms of the studied SPs, with reflections of calcium hydroxide (Ca(OH)_2_, Hydroxyapatite (Ca_10_(PO_4_)_6_(OH)_2_), calcium silicate (Ca_2_SiO_4_), and calcium carbonate (CaCO_3_). Hydroxyapatite mineral takes the most dominant phase at 21–22 two-theta degrees, followed by calcium silicate at approximately 18 two-theta degrees. The mineral phases present are the major constituent of a typical calcined seashell particle [4,10,38]. The result is coherent with the chemical composition data of seashell particles studied by Shavandi et al. [38].

### 3.2. Hardened Properties of Mortar

#### 3.2.1. Direct Compressive Strength Test

**Figure 4:**
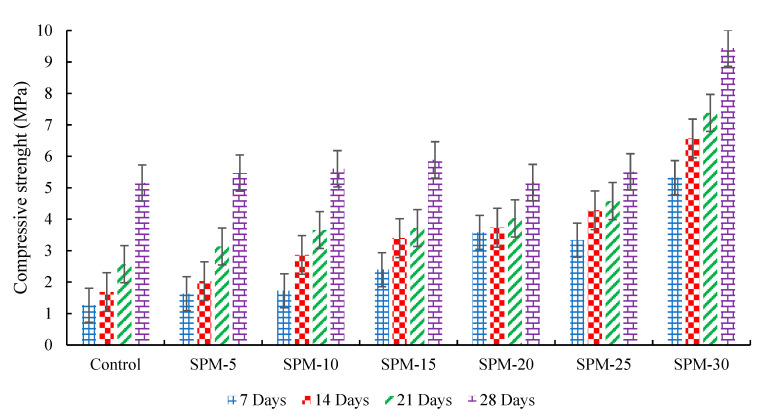
Compressive strength development of SPs-based mortars.

Figure 4 depicts the direct compressive strength measurement of the SP-blended mortar mixtures. The control sample recorded compressive strength values of 1.2 MPa (7 days), 1.66 MPa (14 days), 2.57 MPa (21 days), and 5.15 MPa (28 days), respectively. The mortar’s compressive strength with SPs ranged between 1.63–5.32 MPa for 7 days, 2.03–6.57 MPa for 14 days, 3.13–7.38 MPa for 21 days, and 5.15–9.44 MPa for 28, respectively. These results are entirely dependent on the replacement level and age of hydration. On all the curing days, 5–30% (SPM-5 to SPM-30), the compressive strength development was higher than control. The SPM-30, i.e., the 30% sand replacement with seashell particles, had the best compressive strength development values from all the curing days. There is a noticeable increase of 76.13%, 74.43%, 65.18%, and 45.44% over the control at 7, 14, 21, and 28 days on the SPM-30 design mix. Certainly, as the number of curing days increases, so do the strength values. This result validates cockle and scallop seashell types’ mineral filler effect on improving the hydration of cement [20]. Moreover, CaO in the studied seashells may contribute to the improved compressive strength property of the hardened SP-based mortar blends. CaO is a significant in improving the strength development and density of cementitious products [15]. 

Compared with Suarez-Riera et al. [14], compressive strength results in PC substitution with *Acanthocardia tuberculata* (cockle) seashell type. The authors found that 5% and 15% substitutions reduced compressive strength to 1 and 6%, respectively. Therefore, this study’s findings are marginally at variance with the present study’s findings.

#### 3.2.2. Schmitz Rebound Hammer Test

**Figure 5:**
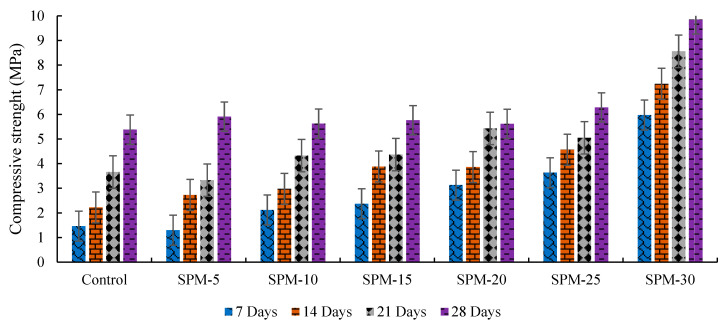
Rebound hammer strength results of SPs-based mortar.

The SP-blended mortar’s surface hardness test determined by rebound hammer strength at seven treatment conditions (0, 5, 10, 15, 20, 25, and 30%) independent of the hydration time up to 28 days is shown in Figure 5, respectively. 

Figure 5 demonstrates that increasing the seashell particle content significantly increases the surface hardness of the SP-modified mortars. For the 7 hydration days, the variation in the strength between 5 to 30% sand replacement is higher than control. For 14 curing days, the rebound hammer strength regularly increased in line with the sand replacement level for all the sand replacements. The 21 days of hydration recorded 3.33, 4.33, 4.37, 5.43, 5.05, and 8.56 MPa for SPM-5 to SPM-30 over the control with 3.566 MPa. The strength value at 28 days continues to improve considerably under all the SPM mixes. Meanwhile, the strength value of the SPM-30 samples increased the most for all the tested samples revealing a ~45.44% increase over the control samples. SPM-5, SPM-15, SPM-20, and SPM-25 recorded increased strength values of ~8.97%, 4.44%, 6.60%, 4.27%, and 14.47% over the control mixture. These findings agree with the filler effect and good adhesion between seashell particles and cement paste in mortar found in the literature [16,19]. 

#### 3.2.3. Ultrasonic Pulse Velocity Test

**Figure 6:**
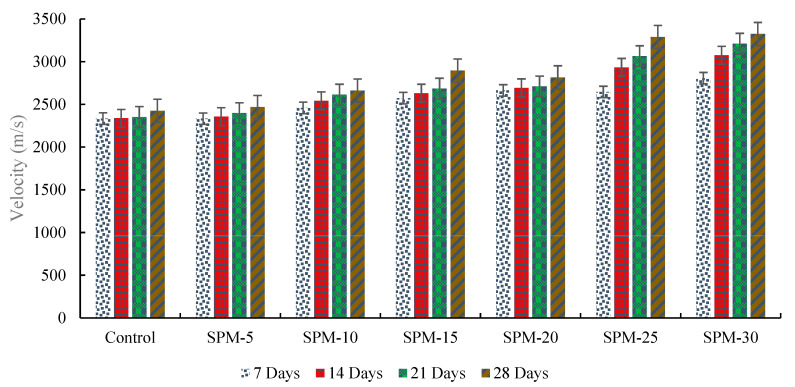
Ultrasonic pulse velocity results of mortars at different treatment conditions with SPs.

Ultrasonic pulse velocity results of mortars at different treatments with SP are presented in Figure 6. The figure detailed SPM-based mixtures’ ultrasonic pulse velocity with different SPs at 7, 14, 21, and 28 days of hydration. It is clear that at 28 days of hydration, the ultrasonic pulse velocity of the mortars with seashells particles is higher than the reference mix due to chemical content and filler effect.

The SPM-30 had the highest ultrasonic pulse velocity values among the mixes induced with a seashell particle. At 7, 14, 21, and 28 days, the ultrasonic pulse velocity of mortars with seashells particles continued to increase more than the reference mix. This phenomenon may be attributed to the higher sand replacement with seashells causing increased hydration in the tested samples. This result is consistent with the direct compression and rebound hammer test results and conforms to the positions of Mo et al. [3,16], Eziefula et al. [4], Suarez-Riera et al. [14], and Tayeh et al. [15] on the and filler effects of a seashell particles on concrete and mortar. The high content of CaO in the SP-blended mortar could initiate the generation of CaCO_3_ arising from infiltration of CO_2_ culminating in the strength development at the later age.

### 3.3. Degree of Hydration Test (Nonevaporable Water)

**Figure 7:**
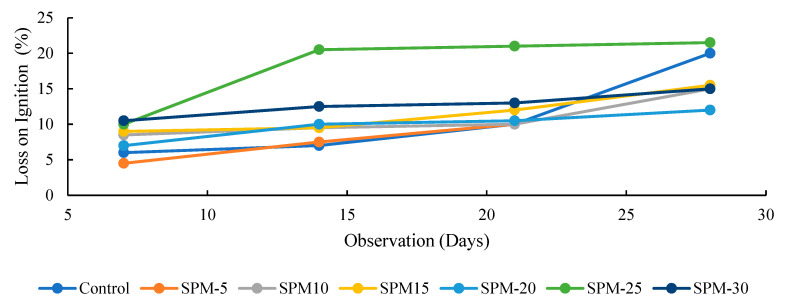
Loss on ignition (LOI) of SP-based mortars.

**Figure 8:**
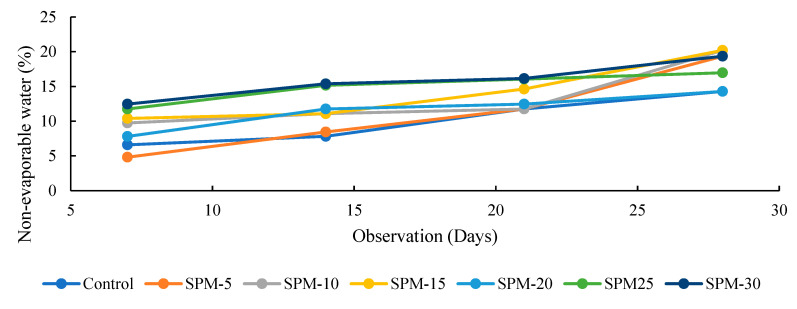
Nonevaporable water (Wn) of SP-based mortars.

**Figure 9:**
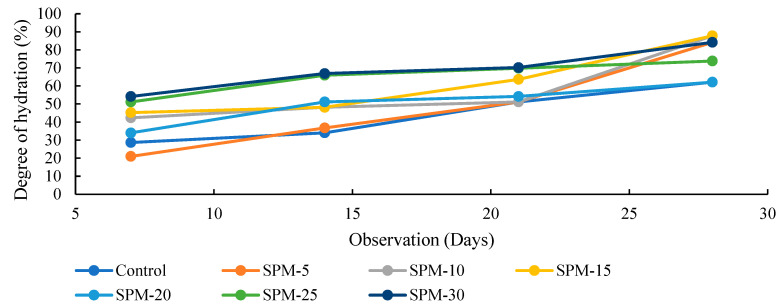
Degree of hydration of SP-based mortars.

Figure 7, Figure 8 and Figure 9 show the loss on ignition, nonevaporable water, and degree of hydration of SP-based mortars determined using the conventional method reported in the literature [36,39] in comparison with the control in 28-day observations. The reported results are calculated according to Equations (2)–(4), taking the loss on ignition of cement and SPs into account. As shown in Figure 7 and Figure 8, the loss on ignition and nonevaporable water of the tested samples increased as the hydration days increased. Moreover, Figure 9 highlights that the degree of hydration increases with hydration time for all SP-blended mortar and control mixes. At 7 days, the hydration rate for all the SP-blended mortars improved, except for the SPM-5 with 5% sand replacement with SPs. The reduced early strength of SP-based mortars could be linked to the presence of CaO in the raw seashell, which could react with the Al_2_O_3_ and gypsum, bringing reduced reaction of the alite. However, the degree of hydration at 28 days increases considerably for the SPs-blended mortar mixes over the control sample. 

Moreover, the degree of hydration for 28 days remains highest in all the SP-modified mixtures, with SPM-30 having the highest value. These results are largely ascribed to the increasing seashell particles in the mixtures. The degree of hydration coincides with the mechanical properties test and confirms the reactive effect of the seashell particles in the mortar mixtures. 

### 3.4. XRD Qualitative Analysis of Hydration Products of Mortar

**Figure 10:**
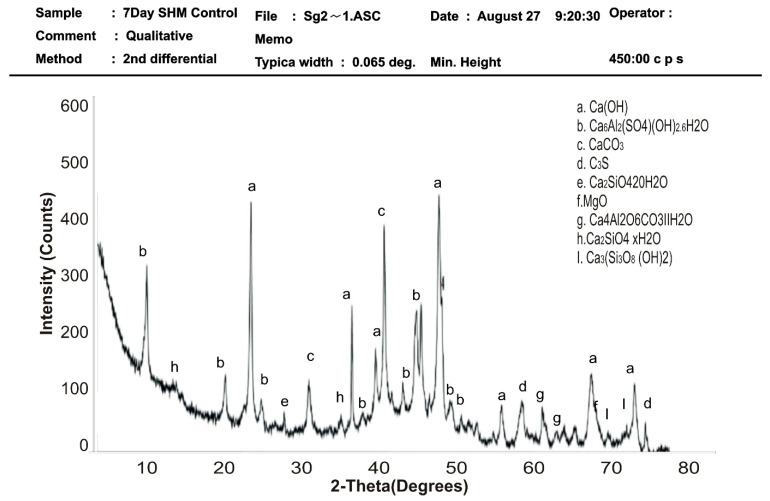
XRD pattern of the hardened control mortar sample at 7 days.

**Figure 11:**
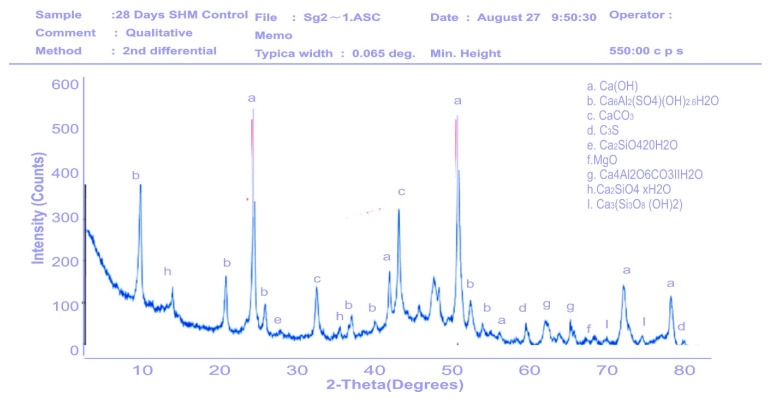
XRD pattern of the hardened control mortar sample at 28 days.

**Figure 12:**
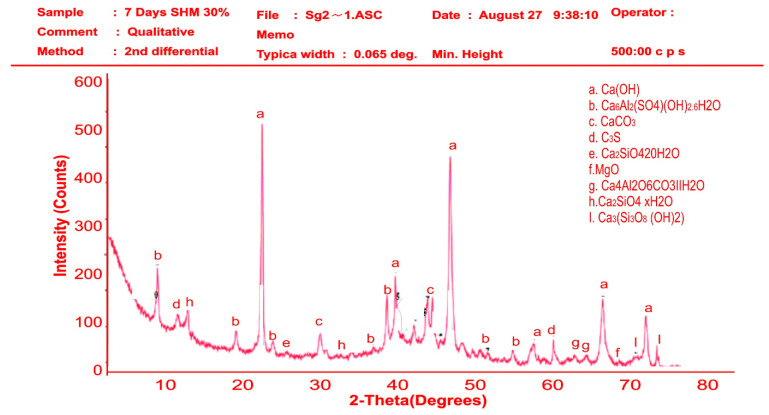
XRD pattern of the SPM-30 sample at 7 days.

**Figure 13:**
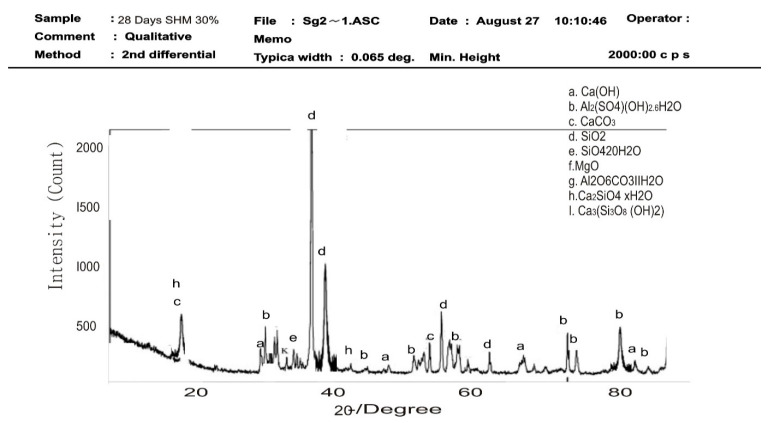
XRD pattern of the SPM-30 sample at 28 days.

The XRD diffraction pattern was used to identify the mineral phases in the hardened mortar samples of control and SPM-30 at 7 and 28 days of hydration. XRD analysis from Figure 10, Figure 11, Figure 12 and Figure 13 presents the XRD patterns of control and SPM-30 mortars. XRD analysis from Figure 10 and Figure 11 presents the XRD patterns of control mortar at 7 and 28 days. From the figure, Ca (OH)_2_, Ca_6_Al_2_(SO_4_) OH_2_6H_2_O, CaCO_3_, Ca_2_SiO_4_20H_2_O, MgO, Ca_4_Al_2_O6CaO_3_11H_2_O, Ca_2_SiO_4_ × H_2_O, and Ca_3_(Si_3_)_8_ (OH)_2_ represented the various mineral phases in plain Portland cement hydrates. From Figure 12, the control sample at 7 days mostly manifested calcium hydroxide (Ca(OH)_2_) at approximately 22 and 48 two-theta degrees of 460 intensity, respectively, followed by calcium carbonate (CaCO_3_) at 40 two-theta degrees of 350 intensity. For the control sample at 28 days, calcium hydroxide (Ca(OH)_2_) peaked at 560 and 530 intensities of 25 and 50 two-theta degrees. This result suggested more reactions of cement and water in the cementitious mortar. Thus, these mineral compositions detected via XRD analysis are typical for a plain Portland cement reaction.

Figure 12 and Figure 13 show the XRD patterns of SHM-30 at 7 and 28 days of hydration. After 7 and 28 days, the same mineral phases observed in Portland cement mortar were detected in SP-blended mortars. After 7 days of hydration, calcium hydroxide peaked at 560 and 530 intensities at 25 and 50 two-theta degrees, followed by ettringite Ca_6_Al_2_(SO_4_) OH_2_6H_2_O mineral. This reaction indicates the slow reaction of a seashell particle in the mortar in the first 7 days of hydration. From Figure 13, SHM-30 at 28 days peaked the most at more than 2000 intensities in 38 and 40 two-theta degrees. Similarly, calcium hydroxide (Ca(OH)_2_) was lower at the peak below 400 intensities. This result further explained the improved mechanical behaviours of mortars replaced with 5 to 30% SPs. The 30% increased seashell particles in the mortar matrix produced more hydration products responsible for the strength enhancement by filling the pore spaces. Thus, with SPs’ filler effect and mineral content, the mixtures’ calcium hydroxide peak intensity with seashell particles was decreased during the 28 days hydration period, especially with the SHM-30 mixture. 

## 4. Conclusions

This experimental study investigates the mechanical and microstructural behaviours of scallop and cockle seashell particles in the blended masonry mortar of a designed 28 days compressive strength of 5.17 MPa. The influence SPs on the prepared blended masonry mortar was analysed through destructive and non-destructive compressive strength tests and advanced XRD technique. The finding of this paper can be summarised as follows:

- The SP (cockle and scallop) particles activated at 600 °C chemically contain calcium oxide (CaO) of about 86%.
- The SEM micrograph of the tested SPs shows mostly irregular and needle-like particles.
- The mineral phases of the calcined seashell particles detected through the XRD technique showed mostly hydroxyapatites, calcium silicate minerals, and calcium carbonate.
- The direct compressive strength investigation showed that with up to 30% sand replacement with SPs in a masonry mortar, an improvement of 45% compressive strength could be achieved compared with control.
- The non-destructive tests conducted using the rebound hammer and ultrasonic pulse velocities machines revealed that replacing sand with SPs in masonry mortar application up to 30% could increase the integrity of the tested masonry mortars over the control.
- The nonevaporable water method of the degree of hydration analysis showed that hydration increased considerably after 28 days for the SP-blended mortars over the control, especially the SHM-30 with 30% sand replacement.
- The SP-blended mortars provided mineral content and filler effect on the microstructure of the masonry mortar matrix, culminating in a reduction in calcium hydroxide in the 28-day XRD peaks of the samples, especially with the SPM-30 mixture.

## Figures and Tables

**Table 1:** Experimental plans.

Type	Experiment	Experimental Factors
Characterisation of the seashells	XRF, SEM, XRD, and BS standard sieving method (PSD)	SPs (scallop and cockle types)
Effect of SPs on PC-based masonry mortar	Compressive strength test (Model YES-2000 Machine)	Substitution ratio of calcined seashell (0, 5, 10, 15, 20, 25, and 30%) Curing ages (7, 14, 21, and 28 days)
	Rebound hammer test (Digital YD225E Rebound Hammer)	Substitution ratio of calcined seashell (0, 5, 10, 15, 20, 25, and 30%) Curing ages (7, 14, 21, and 28 days)
	Ultrasonic pulse velocity test (C269N Pocket Ultrasonic Pulse Velocity Tester)	Substitution ratio of calcined seashell (0, 5, 10, 15, 20, 25, and 30%) Curing ages (7, 14, 21, and 28 days)
	Degree of hydration test(Nonevaporable water method)	Substitution ratio of calcined seashell (0, 5, 10, 15, 20, 25, and 30%) Curing ages (7, 14, 21, and 28 days)
	Microstructure analysis (XRD of selected hardened mortars)	Substitution ratio of calcined seashell (0 and 30%) Curing ages (7 and 28 days)

**Table 2:** Chemical composition of SPs.

Oxides	SiO_2_	Al_2_O_3_	Fe_2_O_3_	CaO	MgO	SO_3_	K_2_O	Na_2_O	P_2_O_5_	MnO	Cl	Sr	LOI
SPs (%)	6.95	2.59	2.40	81.60	3.07	1.20	0.30	0.00	0.55	0.42	0.20	0.50	0.77

**Table 3:** Physical properties of aggregates.

Properties	Sand	SPs
Moisture content (%)	0.63	0.00
Specific gravity	2.70	3.21
Bulk density (kg/m^3^)	1689.96	-
Coefficient of uniformity (Cu)	5.00	3.20
Coefficient of curvature (Cc)	1.80	1.32
Fineness modulus (FM)	3.88	4.06

**Table 4:** Mix constituents of SP-blended mortar.

Constituents	Mix Blends (kg/m^3^)						
	Control	SPM-5	SPM-10	SPM-15	SPM-20	SPM-25	SPM-30
Water	133.72	133.72	133.72	133.72	133.72	133.72	133.72
Portland cement (CEM II 42.5 N)	267.43	267.43	267.43	267.43	267.43	267.43	267.43
SPs	0	70.72	141.43	212.14	282.86	353.57	424.29
River sand (≥300 μm)	1414.29	1343.58	1272.86	1202.15	1131.43	1060.72	989.91

## Data Availability

Not applicable.
